# Selective Human-Milk-Inspired Antimicrobial Peptides for the Treatment of Bacterial Vaginosis

**DOI:** 10.3390/pharmaceutics18030371

**Published:** 2026-03-17

**Authors:** Ishita M. Shah, Carlito B. Lebrilla, J. Bruce German, David A. Mills

**Affiliations:** 1 Foods for Health Institute, Department of Food Science and Technology, University of California Davis, Davis, CA 95616, USA; 2 Matrubials Inc., 630 Pena Drive, Suite 100, Davis, CA 95618, USA; 3 Foods for Health Institute, Department of Chemistry, University of California Davis, Davis, CA 95616, USA

**Keywords:** antimicrobial resistance, bacterial vaginosis, antimicrobial peptides

## Abstract

**Background:** Antimicrobial resistance (AMR) is a global healthcare threat. Traditional largely non-selective antibiotics produce side effects due to the natural host microbiome being modified creating a loss in homeostasis. In women, AMR is a cause of acute generational impact. For example, bacterial vaginosis (BV), the most common gynecological infection in reproductive-age women, is a serious public health concern due to its high rates of recurrence, secondary infections, and reproductive issues; and two currently prescribed antibiotics for BV do not fully resolve the symptoms. **Objective:** The strong need for innovative, potent, safe, and selective therapeutics has prompted a search for such bioactive molecules in milk. Resulting from 200 million years of evolutionary pressure, mammalian lactation not only nourishes infants, but it has also been under relentless Darwinian selective pressure to provide protection from a variety of infections. **Methods:** Computationally designed human-milk-inspired peptides (AMPs) were tested in standard microbicidal assays for activity against BV pathogens, and evaluated for stability and safety. **Results:** Several AMPs are bactericidal towards *Gardnerella vaginalis*, a major BV-associated pathogen, and other BV-associated pathogens. Some novel AMPs do not impact the viability of key lactobacilli linked to a healthy vaginal microbiome. These stable, membrane-acting cationic AMPs reduce inflammation during an infection assay and are safe in EpiVag organoid tissues. **Conclusions:** AMPs can address concerns like non-selectivity and antibiotic resistance—thereby addressing AMR. Lead AMPs from this study offer a promising solution for the development of novel therapeutics for the treatment of BV, which may reduce the burden of AMR.

## 1. Introduction

Bacterial resistance to modern antibiotics is an increasing global health problem. There are few robust and effective models for traditional antibacterial drug discovery, and the discovery pipeline is limited [1]. In addition to the lack of new antimicrobials in development, pathogens continue to evolve multi-drug resistance, and the failure to develop alternative antibacterial therapeutics may have catastrophic consequences [2]. A systematic analysis of the global burden of bacterial resistance in 2019 resulted in a comprehensive estimate of ~1.27 million deaths just in that year [3]. The associated economic burden is high, i.e., ~$3.5 billion per year in excess health care costs (across about 33 countries) and is estimated to cost up to $65 billion in the US by 2050 (CDC, WHO, OECD). Another concern of most currently available, traditional antibiotics is non-specificity, resulting in the elimination of beneficial microbes. Indeed, short-term antibiotic treatments inexorably shift the normal microbiota to dysbiotic states, with potential long-term consequences that aggravate disease and alter digestion, energy metabolism, and importantly, immune pathway regulation [4]. It is increasingly clear that the future of antimicrobial protection must diversify to include alternative approaches, i.e., complex biological macromolecules, including antimicrobial peptides (AMPs), with targeted therapies that fit a distinct safety dossier to reduce the burden of antimicrobial resistance (AMR).

A common, global vaginal infection that impacts women of reproductive age is bacterial vaginosis (BV). Despite its association with important public health issues, including preterm labor and miscarriages, the acquisition and transmission of sexually transmitted infections (STIs) and HIV [5,6], associations with recurrent urinary tract infections (UTIs) [7,8], and pelvic inflammatory disease (PID) [9], the pathogenesis of BV remains unclear, and it has limited treatment options [10,11]. Recurrent BV infections in women can have an impact beyond the individual infection. A major characteristic of BV is the shift from a *Lactobacillus*-dominant, healthy vaginal microbiome, comprising *Lactobacillus crispatus*, *Lactobacillus jensenni* and *Lactobacillus gasseri*, which ensures a vaginal pH < 4.5 and homeostasis. Elimination of these beneficial organisms leads to a dysbiotic condition with an overgrowth of facultative and strict anaerobes and a higher pH [12]. This dramatic shift away from the stable microbial ecology of this specific tissue often leads to the remodeling of the vaginal environment, cascading into multiple adverse outcomes, including secondary infections [13]. BV is characterized by an overrun of polymicrobial biofilms, dominated by *Gardnerella vaginalis* [14] and often, the simultaneous proliferation of bacteria that include *Atopobium vaginae*, *Mobiluncus* spp., *Bacteroides* spp., and *Prevotella* spp. [15], leading to characteristic symptoms such as profuse vaginal discharge and a fishy vaginal odor [16].

The current Standard Of Care (SOC) for BV is common antibiotics, used via oral and topical treatment. These include clindamycin, metronidazole (developed in the 1960s) and tinidazole [17]. Typically, on the day of the visit to the Obstetrics and Gynecology (OBGYN) office, patients are prescribed Metronidazole (500 mg twice-daily oral dose for 7 days or once-daily 5 g vaginal gel of 0.75 and 1.3% *w*/*w* for 5 days) [18,19,20] regardless of information on resistance or sensitivity, but based on fishy odor, pH > 4.5, and the presence of clue cells in a microscopic analysis. All treatment regimens of metronidazole (MTZ), which has broad-spectrum activity against pathogenic anaerobes and a purportedly minimal impact on lactobacilli, result in high BV recurrence rates within 12 months, including patients experiencing recurrence or reinfection [21,22]. Two of the four clades of *G. vaginalis* (Clades 3 and 4) exhibit resistance to metronidazole, with Clade 3 strains all being BV-associated [23]. While short-term cure rates with these antibiotics are equivalent and reach ~80% [24], the reported recurrence rates within 6–12 months are in excess of ~50% [19,21,25]. Although both oral and topical applications of MTZ have a similar efficacy [26], fewer side effects are observed with the vaginal treatment [27,28]. For ~50% patients, when symptoms do not resolve due to resistant pathogens, they are prescribed Clindamycin (300 mg orally or 2% intravaginal application). Clindamycin is non-selective and is known to disrupt the healthy vaginal microbiome and the intestinal microbiome. Nonetheless, Clindamycin is unable to fully eradicate the vaginal biofilms, thus resulting in high recurrence rates of BV. Orally administered clindamycin impacts the gut microbiome to such an extent that many patients feel they have diarrheal disease (~3 months post-antibiotic administration) and prefer to bear the pain of BV rather than take the medication. Antibiotic treatment for BV with the side effect of disrupting the intestinal microbiome can severely precipitate colonization with *Clostridium difficile* [29]. Thus, alternative local antiseptic treatment options have been considered; however, the efficacy of prolonged and repeated vaginal antiseptic applications has been low. Some additional examples include lactic acid-containing vaginal gels. These are shown to be effective in the prophylaxis of BV [30]. However, these semi-solid gels are messy to apply (resulting in poor patient compliance), prone to leakage (thus needing frequent administration), and their therapeutic efficacy is often limited due to their short residence time in the vagina [31]. An additional therapy involves the use of octenidine, with a cure rate of 62.5%, but this still leads to complete resistance in 37.5% of patients [32]. In short, current BV therapies are not sufficient to address the disorder, and novel strategies are required to overcome the high recurrence and relapse rates associated with BV [17]. Antimicrobial therapeutics that have broad activity against BV pathogens while simultaneously preserving the healthy *Lactobacillus*-dominant microbiome would represent a major advance in the treatment of BV.

A strategy to find new antimicrobials that are simultaneously effective, selective, and safe is to embrace milk—a substance known to protect mothers and infants from bacterial infections [33]. Milk provides essential nutrients for growth, provides passive and adaptive immunity, and regulates the developing infant gut microbiota. Research communities around the world have studied the diverse components of the bioactive molecules in milk. The University of California (UC) Davis Milk Bioactives Program has recently characterized a subset of important milk components: peptides. The digestion of milk proteins begins in the mammary gland, producing thousands of naturally occurring peptides [34]. Milk peptides are released by a complex system of endogenous proteases, anti-proteases, and protease activators within the mammary gland. Using state-of-the-art mass spectrometry, thousands of peptides have been isolated and identified in freshly expressed human and mammalian milks [34,35,36]. Importantly, some fractions of the peptide pool exhibit antimicrobial activity [34]. For research purposes, human milk can be made available through donor milk banks and fractionated into peptide pools. However, for clinical development and translation, the use of donor milk and flash fractionation chromatography, which can result in some batch-to-batch variation among the pools of peptides, is not scalable. Therefore, we subsequently evaluated the activity of synthetic peptides, as these can be manufactured in gram and kilogram quantities for the development of active pharmaceutical ingredients (APIs). In this work, we show that when tested, a group of synthetic peptides encoded within human β-casein, αS1-casein, lactoferrin, lactase, and variants thereof demonstrated antimicrobial function and therapeutic potential. Hence, these peptides are referred to as milk AMPs. In standardized microbicidal assays, these milk AMPs act rapidly against many clinically relevant BV pathogens. Importantly, *G. vaginalis*, a key pathogen associated with BV, is susceptible to these milk peptides, whereas the various *Lactobacillus* species that dominate a healthy vaginal microbiome are naturally resistant to these milk AMPs. Furthermore, these milk AMPs render other BV-associated pathogens non-viable. This unique selectivity positions these AMPs as promising therapeutic agents for eliminating pathogens associated with BV without causing a disruption of indigenous *Lactobacillus* communities. Additionally, these AMPs have been deemed safe and do not promote the emergence of resistance. Thus, instead of using approved or modified antibiotics as repurposed drugs, which can still have side effects due to non-selectivity and can harm the healthy vaginal microbiome and promote the emergence of resistance, AMPs may be innovative, potent and safe drugs. In work to be described in a companion paper, collaborators at Contraceptive Research and Development (CONRAD), Eastern Virginia Medical School (EVMS) have evaluated the impact of these AMPs on viruses associated with STIs.

## 2. Materials and Methods

### 2.1. Bacterial Strains and Media

Pathogenic bacteria were obtained from the American Type Culture Collection (ATCC, Manassas, VA, USA) and the Biodefense and Emerging Infections Research Resources Repository (BEI Resources, Manassas, VA, USA). The *G. vaginalis* strains used in our study are as follows: ATCC (14018) and BEI resources (HM-1105, HM-1106, HM-1107, HM-1110 and HM-1112). *G. vaginalis* was typically grown on Human Blood-Tween 80 (HBT) bilayer media (Remel, Thermofisher, Waltham, MA, USA), and liquid cultures were grown in Tryptic soy broth (TSB) supplemented with 10% horse serum (Sigma Aldrich, St. Louis, MO, USA). Other pathogens obtained from ATCC were *Atopobium vaginae* (BAA-55), grown on Tryptic Soy agar with 5% defibrinated sheep blood, *Mageeibacillus indolicus* (BAA-2120), grown on Brucella agar with 5% defibrinated sheep blood, *Mobiluncus curtusii* (35241), grown on Tryptic Soy agar with 5% defibrinated sheep blood, and *Lactobacillus iners* (BAA-3226), grown on Tryptic Soy agar with 5% defibrinated sheep blood. These pathogens were grown anaerobically. *Lactobacillus crispatus*, *Lactobacillus gasseri* and *Lactobacillus jensenii* were retrieved from the UC Davis Food Science and Technology Biobank collection, maintained on De Man, Rogosa, and Sharpe (MRS) agar, and grown in MRS broth anaerobically.

### 2.2. Impact of AMPs on BV Pathogens and Commensal Lactobacilli

*G. vaginalis* strains were evaluated against a total of 28 peptides. These peptides were designed from human β-casein, α-S1 casein, lactase and lactoferrin to meet specific criteria relevant to length, with a positive net charge at pH 7 and predicted water solubility via selection from the indicated human milk proteins. The parent sequence was used to incorporate minor amino acid substitutions based on predictions of improved efficacy. Standardized antibiotic susceptibility testing using CLSI methods to determine MBC values were employed [37], and finally, four peptides were selected for the subsequent work: UCD-MAT-001 (12-amino acid peptide encoded within human β-casein, with four amino acid substitutions compared to the parent sequence, net charge at pH 7: +5), UCD-MAT-002 (10 amino acid peptide encoded within human α-S1 casein, 2 amino acid substitutions compared to the parent sequence, net charge at pH 7: +4), MAT-006 (10 amino acid peptide encoded within human lactase, 1 amino acid substitution compared to the parent sequence, net charge at pH 7: +4) and MAT-014 (11 amino acid peptide encoded within human lactoferrin, 2 amino acid substitutions compared to the parent sequence, net charge at pH 7: +3.9). These were used for continued preclinical efficacy and safety screening. These peptides were obtained from Genscript, Piscataway, NJ, USA (MS, HPLC and COA files available in Supplementary Material), and stock solutions were stored frozen at 100 mg/mL in water until ready for use. For the minimum bactericidal concentration (MBC) evaluation, we used diluted TSB/horse serum media, as has been a useful strategy for numerous AMPs in previous studies, where a 1:100 dilution of standard Mueller–Hinton broth (MHB) or 1:100 Brain Heart Infusion (BHI) broth dramatically improves efficacy [37]. For easy evaluation, the inoculum concentration used was ~1 × 10^6^ CFU/mL. Assessment of the effects of the peptides on additional pathogens and beneficial lactobacilli included short-term time-kill assays using 0.8% NaCl as the diluent for time-dependent loss in viability, and to decipher minor differences between individual peptides. Experiments were performed in triplicate to include biological and technical replicates.

### 2.3. Evaluation of AMP Folding

The four peptides were thawed to room temperature (RT) and diluted to a final concentration of 0.5 mg/mL in either 0.01X Tryptic soy broth with horse serum (TSB.HS) or 0.8% NaCl to determine the impact of diluents on folding. These samples and the diluent controls were submitted to the UC Davis circular dichroism (CD) core facility. CD spectra were recorded using the Jasco J715 CD spectrometer (Tokyo, Japan), and upon subtracting the values obtained for the diluent control, the data were plotted using Prism and analyzed for protein folding based on the shape of the curve [38].

### 2.4. Selective Activity Towards BV Pathogens in Co-Cultures

In vitro co-cultures were set up by combining BV pathogens and healthy vaginal bacteria with an inoculum ratio of 1:1. Adapted from [39], for pathogens, *G. vaginalis* was used at 85% to represent dominance (0.42 × 10^6^ CFU/mL), whereas the three other BV pathogens were used at 5% each (~0.25 × 10^5^ CFU/mL), and for healthy bacteria, *L. crispatus* represented 85% of the total (0.42 × 10^6^ CFU/mL), and the remainder were equal % of *L. gasseri* and *L. jensenii* (~0.38 × 10^5^ CFU/mL). The four AMPs, UCD-MAT-001, UCD-MAT-002, MAT-006 and MAT-014, were tested at a final concentration of 0.15 mg/mL each, using 0.8% NaCl as a diluent along with simulated vaginal fluid (BioChemazone, Leduc, AB, Canada) in a final volume of 1 mL, and 3 μL aliquots were spotted at T0, T2 and T6 h post-addition of AMP onto an HBT bilayer, TSA with 5% defibrinated sheep blood, Brucella Agar with 5% sheep blood, and MRS agar. Plates were incubated in the anaerobic chamber, and growth was scored after 48 h.

### 2.5. Fluorescence Assays for Membrane Activity of AMPs

For the mechanism of the membrane activity of these AMPs, we evaluated N-Phenyl-1-napthylamine (NPN) uptake (for membrane permeabilization at a 10 μM final concentration) and Bis-(1,3-Dibutylbarbituric Acid) trimethine Oxonol (DiBac_4_(3)) uptake (for membrane depolarization) as per standardized methods [40]. Briefly, upon incubation of AMPs with bacteria with an inoculum of 1 × 10^6^ CFU/mL for 2 h at 1/2X of the MBC, to assess the membrane permeabilization of *G. vaginalis* and *L. iners*, NPN was added to each well, and fluorescence units were recorded at Ex/Em λ = 360 nm/λ = 450 nm. In the data processing workflow, the fluorescence units resulting from wells with bacteria but without AMP treatment were subtracted for normalization. To assess the cytoplasmic membrane depolarization activity, DiBac_4_(3) was used at 0.4 μM, and fluorescence was measured using Ex/Em λ = 485 nm/λ = 528 nm. Here as well, in the data processing workflow, the fluorescence units resulting from wells with bacteria but without AMP treatment were subtracted for normalization. These experiments used a cationic AMP, Indolicidin [41], as a positive control, and a BioTek Synergy 2 (Agilent, Santa Clara, CA, USA) plate reader for fluorescence measurements.

### 2.6. Biofilm Disruption Activity of AMPs

Biofilms of *G. vaginalis* 14018 were established by incubating 1 × 10^6^ CFU/mL in 96-well plates and allowing growth for 48 h at 37 °C in the presence of 5% CO_2_. Upon centrifugation at 1000 rpm and removal of supernatant, subsequently, biofilms were subjected to increasing concentrations of AMPs for 1 h prior to staining with 0.1% crystal violet, extraction using 33% acetone, and measurement at λ = 630 nm [42].

### 2.7. Evaluation of Rate of Resistance Emergence

Using standardized methodology for determination of resistance against an antibacterial agent [43], 1 × 10^8^ CFU/mL *G. vaginalis* 14018, *A. vaginae* and *M. curtusii* were either left untreated in broth or treated with AMPs (starting with 1/20th MBC of either 32 or 16 μg/mL, depending on the AMP). For *G. vaginalis* 14018, Metronidazole was used as a control. With 1:100 dilutions after O/N incubation through the generations to reach MBC, aliquots were serially diluted, and CFU/mL was calculated. Resistance was calculated based on CFU/mL counts in the culture without the AMP.

### 2.8. AMP Stability Determination

The AMP resuspensions (100 mg/mL stocks) were stored at −20 °C, as is typical. For stability measurements, alterations in efficacy, if any, were monitored after subjecting the AMPs to specific conditions: aliquots of the AMPs were incubated at room temperature (RT) for 2 weeks, 1 month, and 3 months, and at 42 °C and 70 °C for 1 h prior to efficacy testing for *G. vaginalis* using 0.8% NaCl as the diluent and the standard time-kill assay, adapted from [44].

### 2.9. Cytotoxicity Studies

Using monolayers of vaginal epithelial cells, VK2/E6E7, established in DMEM/10% FBS, and cervical epithelial cells and HeLa (ATCC, CRM-CCL-2) established in EMEM/10% FBS, and an initial seeding density of 5 × 10^4^ cells/mL, we incubated the AMPs in a dose-dependent manner (1–10 mg/mL) for 36 h prior to measurement of cellular viability via Calcein dye (L3224, Invitrogen, Carlsbad, CA, USA), as per the manufacturer’s instructions, and excitation λ = 485 nm, emission λ = 528 nm). For comparison, we treated cells with a cytotoxic agent, such as ethanol (70%), for 1 h to determine the change in fluorescence units.

### 2.10. MTT Assay for ET_50_ Determination Using EpiVag Organoids

AMPs were evaluated for their impact on EpiVag organoid models (MATTEK, Ashland, MA, USA) using a standard MTT time course assay [45] according to the manufacturer’s instructions. Briefly, the AMPs were prepared in 20 mg/mL solutions for this assay. Sterile, deionized water was used as a negative control, and 1% Triton X-100 was used as a positive control. Upon preparation of the tissues, the experiment was initiated by the addition of 100 μL of the AMP solution or a control directly on top of the tissue and was then incubated for an appropriate exposure time prior to assessment of MTT reduction via absorbance measurement at λ = 500 nm using a Molecular Devices VersaMax Plate reader (San Jose, CA, USA). The test validity was established by ensuring that treatment with Triton resulted in the expected ET_50_ value (~1.5 h in this setup). This assay was conducted by IIVS, Gaithersburg, MD, USA.

### 2.11. In Vitro Infection Assay and Impact on Inflammation

VK2/E6 cells were obtained from ATCC (CRL-2616) and were propagated using the manufacturer’s instructions in DMEM with 10% FBS. For the infection assay, adapted from [46], monolayers of VK2/E6E7 were established in 24-well plates at a seeding density of 5 × 10^4^ cells/mL by seeding the plates 2 days prior to the assay. *G. vaginalis* was grown overnight in TSB.HS media, inoculated at a 1:100 ratio in fresh media, and grown for 2 h prior to washing and resuspending in 1X PBS. Monolayers were also washed once with media. Half of the 24-well plate was treated with *G. vaginalis* at a multiplicity of infection (MOI) of 10, and after 2 h of infection, supernatants were removed, and fresh media was added to the wells. Then, AMPs were added at a final concentration of 0.5 mg/mL to ensure that the triplicate wells represented a no-treatment control, AMP-only controls, *G. vaginalis*-only control, and *G. vaginalis* in combination with the AMPs. The plates were then incubated at 37 °C in the presence of 5% CO_2_ for 24 h prior to aspirating the supernatant and treating the monolayers in each well with 0.75 mL of Trizol (Thermofisher) for subsequent RNA isolation using a chloroform/isopropanol extraction process. The resulting RNA pellet was rehydrated in ultrapure water at 56 °C for 30 min and treated with DNase I (AM1906, Invitrogen) for 30 min at 37 °C prior to enzyme neutralization at RT for 5 min. Subsequent cDNA synthesis was initiated using a high-capacity cDNA reverse transcription kit (4368814, Applied Biosystems, South San Francisco, CA, USA), and qPCR was carried out using FAST SYBR Green (4385612, Applied Biosystems). Using Actin as a control, the induction ratio was measured for inflammatory markers like IL-6, IL-8, TNFα and IL-1β. The primer sequences are as follows:

-Actin F, 5′-CACCATTGGCAATGAGCGGTTC-3′;

-Actin R, 5′-AGGTCTTTGCGGATGTCCACGT-3′;

IL-6 F, 5′-AGACAGCCACTCACCTCTTCAG-3′;

IL-6 R, 5′-TTCTGCCAGTGCCTCTTTGCTG-3′,

IL-8 F, 5′-GAGAGTGATTGAGAGTGGACCAC-3′;

IL-8 R, 5′-CACAACCCTCTGCACCCAGTTT-3′;

TNFα F, 5′-CTCTTCTGCCTGCTGCACTTTG-3′,

TNFα R, 5′-ATGGGCTACAGGCTTGTCACTC-3′;

IL-1β F, 5′-CCACAGACCTTCCAGGAGAATG-3′; and

IL-1β R, 5′-GTGCAGTTCAGTGATCGTACAGG-3′.

## 3. Results

### 3.1. Selective Bactericidal Activity of Human-Milk-Inspired AMPs on Pathogens Associated with Bacterial Vaginosis

Synthetic peptides, designed computationally to incorporate features that could predict efficacy against bacterial membranes, were evaluated for their ability to show desirable MIC and MBC values for pathogens. Peptides with a length of 10–12 amino acids and a net charge at pH 7 ranging from +2 through +5 were included in the screening process. Milk-inspired AMPs are efficacious in diluted media, which has been a useful strategy with numerous peptides in previous studies (0.01X MHB or 0.01X BHI broth), as described in detail in [37] for numerous AMPs. The peptides were first evaluated for activity against standard ESKAPE pathogens, such as *Pseudomonas aeruginosa,* and revealed dramatic differences upon resuspending the AMPs in diluted MHB (MBC ~8–16 μg/mL). This difference in activity was matched with solution structure by using CD spectroscopy to reveal random coil structures in solution (when in diluted MHB). The same method was used for testing AMPs against BV pathogens, and again, it was observed that diluting standard TSB (+10% horse serum) was better able to predict the efficacy of these peptides against *G. vaginalis*. Of the four selected AMPs that showed efficacy, a range of MBC (Minimal Bactericidal Concentration) values from 8 μg/mL to up to 32 μg/mL, depending on the BV pathogen and the AMP, was observed (Table 1, top panel). A common antibiotic used for bacterial vaginosis, Metronidazole, served as a positive control in these experiments.

**Figure 1:**
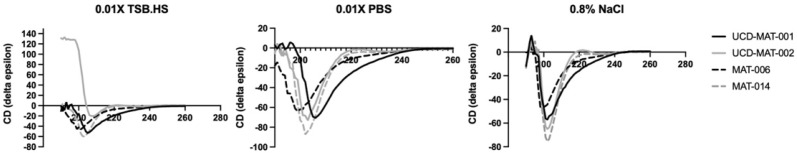
CD spectra of peptides in different resuspension buffers. As a way of determining the impact of resuspension buffers on future formulations, peptide activity was tested in 0.01X TSB.HS (TSB with Horse serum), 0.01X PBS and 0.8% NaCl. While UCD-MAT-002 is as bactericidal as MAT-006 in most cases in 0.01X TSB.HS, in 0.01X PBS and 0.8% NaCl, MAT-006 appeared to be the most effective in time-kill assays.

To further differentiate between individual candidate AMPs, short-term time-kill assays using additional resuspension buffers (0.01X PBS and 0.8% NaCl) to determine the time-dependent loss in viability of other BV pathogens were performed. It was observed that 0.8% NaCl allows for better efficacy of these four AMPs compared to PBS-based solutions, and this method was used for the evaluation of their efficacy against *A. vaginae*, *M. indolicus* and *M. curtusii*. MBC, in each case, was determined by spotting aliquots at times T0 h, 1 h, 2 h, 4 h and 8 h (Table 1, bottom panel). Importantly, it was noted that, while at T4 h, MAT-006 and UCD-MAT-002 appear to work equivalently well, MAT-006 is more efficient at earlier times, indicating that MAT-006 is a better candidate overall for targeting BV. While UCD-MAT-002 is as bactericidal as MAT-006 in most cases in 0.01X TSB.HS, in 0.01X PBS and 0.8% NaCl, MAT-006 appeared to be the most effective in the time-kill assays. Additional tests with healthy vaginal lactobacilli (*L. crispatus* and *L. jensenii*) showed selective activity of UCD-MAT-001, UCD-MAT-002 and MAT-006. Unlike other AMPs, which did not result in a loss of viability of the lactobacilli at concentrations as high as 5 mg/mL, MAT-014 appeared to be bactericidal towards these beneficial bacteria. Interestingly, *L. iners* is also sensitive to these AMPs. Amongst vaginal lactobacilli, *L. iners* has the smallest genome, and while it is commonly present in a healthy vagina, it is often recovered in BV and colonizes after a disturbance in the vaginal environment, offering overall less protection against vaginal dysbiosis and subsequently leading to BV and other complications [47]. In comparison to that, other *lactobacilli* associated with the healthy vaginal microbiome are resistant to three of the four AMPs (Table 1, bottom panel), suggesting selective activity of some of these AMPs. During disease states, the vaginal microbiome is dominated by pathogens, so the selective activity of AMPs against pathogens can be of use, as that can still ensure the viability of lactobacilli, which can grow once the vaginal epithelium has recovered from the infection. It has long been known that commensal bacteria like lactobacilli mostly have saturated fatty acids in their membrane structure, with straight, unkinked tails, thus decreasing bilayer fluidity. Higher concentrations of saturated fatty acids in the membrane confer AMP resistance [48], and this could be the case for these milk AMPs. To correlate this activity with their secondary structures in solution, the AMPs were subjected to CD spectra analysis in the indicated resuspension buffers (Figure 1), which indicated random-coil structures.

To establish their efficacy against bacteria in a mixed culture, AMPs were evaluated for differential elimination of pathogens in co-cultures as a way of simulating the initial stages of colonization by BV pathogens. Here, the viability of BV pathogens in the co-cultures were monitored through their growth on the HBT bilayer, TSA with 5% defibrinated sheep blood, and Brucella agar with 5% defibrinated sheep blood, whereas the viability of lactobacilli was monitored on MRS agar. Whether NaCl or simulated vaginal fluid were used as the base for these assays, it was observed that when using UCD-MAT-001, UCD-MAT-002 and MAT-006, a loss of viability was noted only for pathogens, but not for *lactobacilli*, whereas for MAT-014, there was a loss in CFU on the MRS agar plates, indicating alterations in the viability of lactobacilli (Table 2).

To evaluate the efficacy of individual AMPs or combinations, and to maximize the potential of selecting one or two AMPs from these four for further evaluation, all four AMPs were included in subsequent assays to test whether any of these AMPs showed subsequent difficulty regarding stability, resistance or subsequent translation from in vitro efficacy to ex vivo and in vivo safety.

### 3.2. Mechanism of Action of AMPs

**Figure 2:**
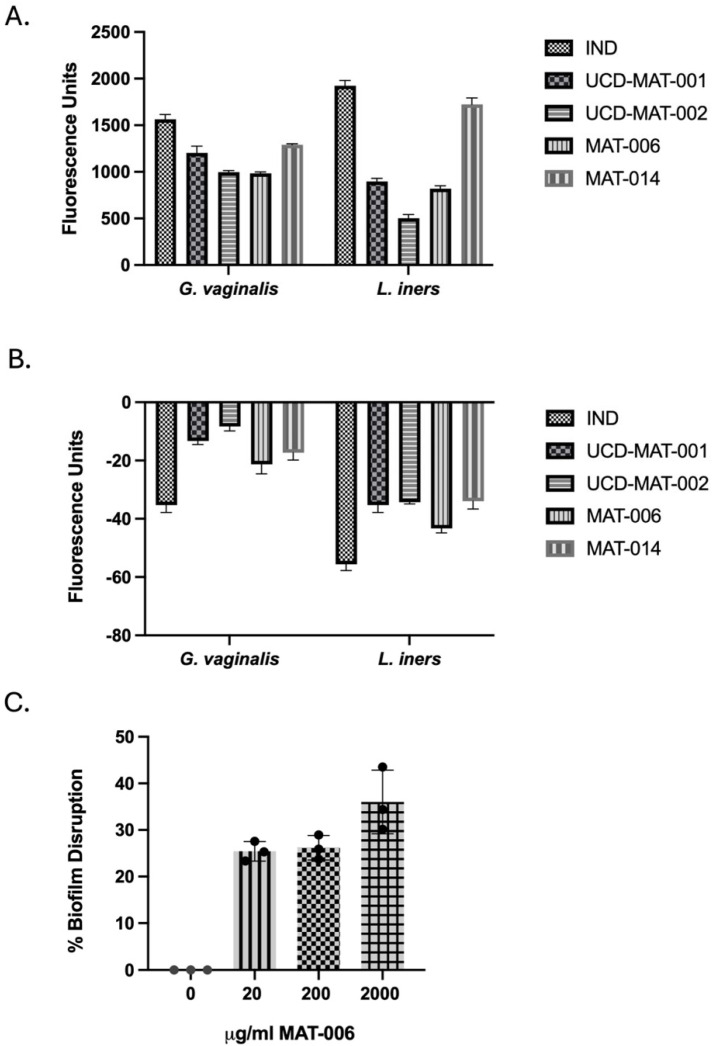
Mechanism of action of AMPs. (**A**) Measurement of membrane permeabilization using the standard NPN assay for all four AMPs, where an increase in fluorescence indicates NPN uptake by the cell. (**B**) Measurement of changes in cytoplasmic membrane polarization due to fluorescence units changing in the presence of DiBac_4_(3) dye for all four AMPs. Bar graphs represent the mean with SD. A previously characterized AMP, Indolicidin (IND), was used as a positive control. (**C**) Biofilm disruption of the major BV pathogen *G. vaginalis* within 1 h with MAT-006 at indicated concentrations, evaluated using Crystal violet staining. The scatter dot plot represents the mean with SD.

To establish the mechanism of action in the disruption of membrane activity of these cationic AMPs, studies evaluated NPN uptake for membrane permeabilization and DiBac uptake for membrane depolarization, as per standardized methods [40]. All four AMPs permeabilized the *G. vaginalis* and *L. iners* membranes, as observed by an alteration in fluorescence units (excitation λ = 360 nm, emission λ = 450 nm) compared to cells without AMP treatment, which is corrected to 0 (Figure 2A). For the cytoplasmic membrane’s depolarization activity, it is known that a decrease in fluorescence is observed due to hyperpolarization, where the dye leaves the cell, and its signal decreases [49]. Here, using DiBac_4_(3), we demonstrate that all four AMPs caused hyperpolarization of the cytoplasmic membrane (Figure 2B).

To determine if these AMPs can indeed disrupt biofilms that are associated with BV, *G. vaginalis* biofilms were subjected to increasing concentrations of AMPs (MAT-006 shown here) for 1 h prior to staining and fluorescence measurements. Concentration as low as 20 μg/mL (MBC) was able to show a reduction in the biofilm within one hour (Figure 2C).

### 3.3. Rate of Resistance Emergence

Using MAT-006, the rate of the emergence of resistance for select BV pathogens was evaluated, starting at 1/20th of the MBC and serial passaging through generations after overnight incubation to reach MBC [43]. Resistance was calculated based on CFU counts for the culture in the absence of the AMP. As was expected of the AMPs [50], based on the CFU counts, we conclude that the rate of the emergence of resistance against MAT-006 (shown here) was low and was not detectable in our assays (Table 3). For *G. vaginalis*, Metronidazole was used as a control in the assay and showed an emergence of resistance at a rate of ≤1/1.2 × 10^7^, demonstrating the superiority of MAT-006 over Metronidazole in this assay.

### 3.4. Stability of AMPs

For extensive evaluation and subsequent translation of any AMP, stability plays a crucial role. Changes in temperature and the impact of host factors can alter the solution structure of an AMP, which, in turn, can alter its biological function, thus limiting the use of such an AMP. We assayed for AMP stability via storage at different temperatures, with evaluation of MBC as a marker for function, and observed that these AMPs remained stable in solution and did not lose their bactericidal properties upon temperature shifts (Table 4). This information is critical for the next steps of therapeutic development, including formulation and storage for additional in vivo assays.

### 3.5. Safety Evaluation of AMPs

**Figure 3:**
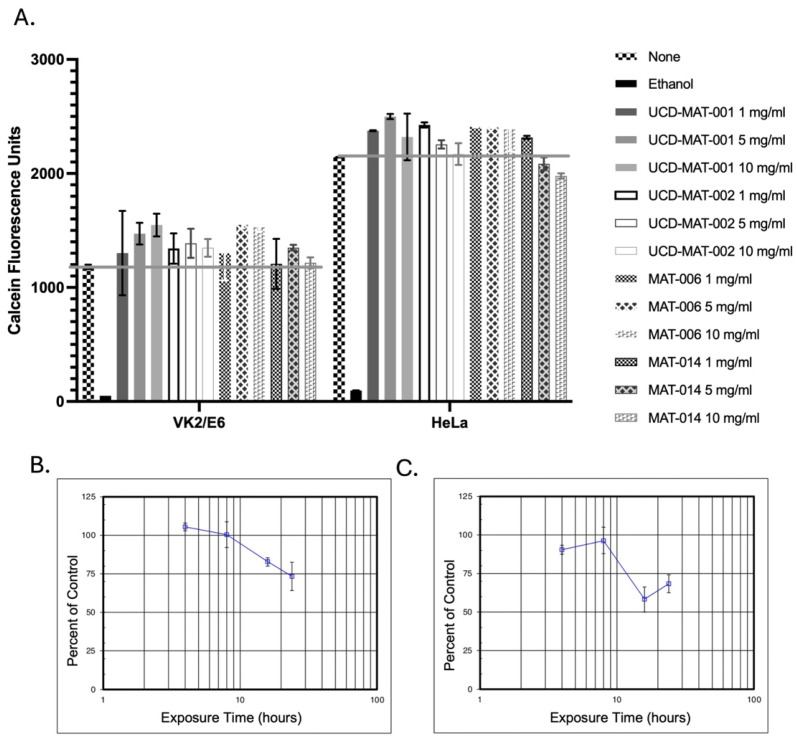
Safety evaluation of AMPs. (**A**) Cytotoxicity assays using monolayers of VK2/E6E7 and HeLa cells using fluorescence measurements with Calcein dye following the treatment with AMPs in a dose-dependent manner, using “None” as a no-treatment control, and ethanol as a positive control. The bar graph represents the mean with SD from triplicate samples. The grey line indicates measurements for “None” in the assay for ease of comparative visual evaluation. (**B**) MTT assay with human EpiVag tissues and UCD-MAT-002 (20 mg/mL). (**C**) MTT assay with human EpiVag tissues and MAT-006 (20 mg/mL).

The safety profile of these AMPs was evaluated via standard cell lines as well as organoids. We used monolayers of vaginal epithelial cells, VK2/E6E7, and cervical epithelial cells, HeLa, and incubated the AMPs in a dose-dependent manner (1, 5 and 10 mg/mL) for 36 h prior to measurement of cellular viability using permeability to Calcein dye. Compared to treatment of cells with ethanol (used here as a control), results showed that the presence of AMPs with the host cells caused no loss in host cell viability at concentrations up to 10 mg/mL, much higher than the effective MBC dose, thus offering a promising in vitro therapeutic index (Figure 3A). As such, these data are in agreement with AMPs being selectively active against negatively charged membranes of microbes while leaving the membranes of eukaryotic cells, which are composed of uncharged neutral phospholipids, sphingomyelins, and cholesterol, unharmed [51]. Moreover, these peptides were tested at a 2-fold higher concentration (i.e., 20 mg/mL final) in the MATTEK EpiVag tests using human vaginal epithelium and a standard MTT time course assay. Here, all four AMPs resulted in an ET_50_ > 24 h, which is higher than some other currently available vaginal products, and is higher compared to the assay positive control, Triton, which results in an ET_50_ of ~1.5 h. Figure 3B,C show the safety of UCD-MAT-002 and MAT-006, respectively. These data thus strengthen the safety dossier of milk-inspired AMPs.

### 3.6. Impact on Inflammation During an In Vitro Infection Assay with G. vaginalis

**Figure 4:**
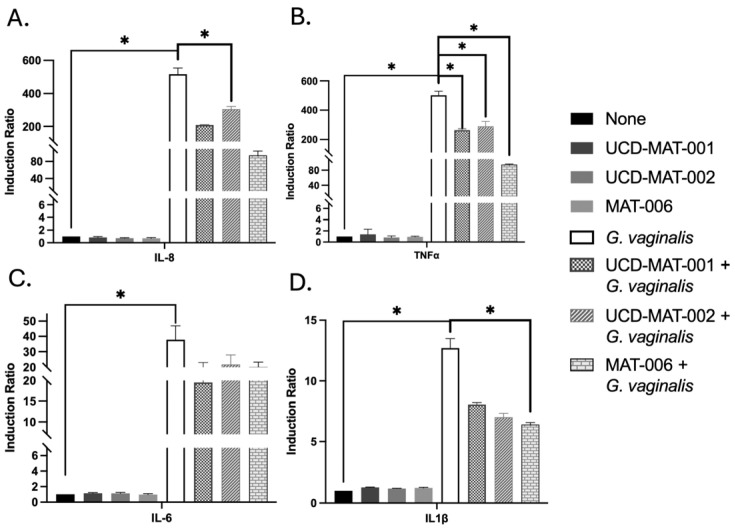
Impact on inflammation during an in vitro infection assay with *G. vaginalis*. Effects of AMPs on VK2/E6E7 cells in the absence and presence of *G. vaginalis* infection. (**A**) Induction ratio of IL-8. (**B**) Induction ratio of TNFα. (**C**) Induction ratio of IL-6. (**D**) Induction ratio of IL-1β. *p*-value as * is indicated where significantly different (*p* < 0.05) from paired *t*-test (two-tailed) analysis.

During an infection of VK2/E6E7 monolayers with *G. vaginalis*, using RNA and qPCR, the effects of subsequent AMP incubation on inflammatory markers like IL-6, IL-8, TNFα and IL-1β were measured. Herein, UCD-MAT-001, UCD-MAT-002 and MAT-006 were used at a concentration well above the in vitro MBC, using the knowledge of the in vitro safety of vaginal epithelial cells in the presence of AMPs. This assay shows that the presence of AMPs resulted in reduced levels of expression of these markers during the infection, as evidenced by comparing the levels in the presence of *G. vaginalis* alone versus in combination with AMPs. This reduction in the induction ratio, in turn, indicates a suppression of the infectious and inflammatory responses elicited by *G. vaginalis* (Figure 4). Importantly, in the absence of *G. vaginalis*, the AMPs do not have any impact on these markers, broadening their safety dossier and their overall beneficial support for the host cells.

## 4. Discussion

AMR has been declared by the WHO to be one of the top 10 global public health threats. The evolution of such resistance is driven by numerous, complex mechanisms, both biological and societal in nature, resulting in the increased spread of diseases and, inevitably, increased death. Thus, “AMR’ and “superbugs” have become so endemic to modern medicine that they have become commonly used terms. Importantly, in addition to just being a treatment issue in infectious diseases, AMR increases the risks of numerous routine medical procedures, as well as major surgeries, and for patients undergoing transplants or chemotherapy. Other underlying disease conditions often predispose immunocompromised patients to microbial infections. Due to there being few robust and effective models for traditional antibacterial drug discovery [1], there has been a catastrophic lack of new therapies in development [2,52], with a continued rise in pathogens that are becoming multi-drug resistant. Furthermore, in 2017, there were 31 products working against priority pathogens in development. An annual analysis revealed a decline in 2021, with 27 new antibiotics in development. Overall, the situation is grim, needs urgent attention, and will require innovation not just at the bench, but in regulatory agencies, in boardrooms, and in political discussions. The use of traditional antibiotics is complicated. Firstly, these antibiotics have a limited lifespan because, on average, once a new antibiotic is introduced in patient communities, resistance starts to emerge within 8–10 years, and secondly, most currently available antibiotics are non-selective, such that they harm beneficial bacteria associated with health. As a result of the elimination of the natural, protective microbial community, even a single round of an antibiotic regimen, within a year, often shifts the normal microbiota to a dysbiotic state, resulting in longer-term clinical complications. Thus, it is indeed clear that the future of recovery from infections should use a multi-prong approach that can use diverse antimicrobial modalities and alternative approaches. In women, specifically, recurrent gynecological infections like BV, if left untreated, result in an umbrella of secondary complications, including a debilitating impact on newborns.

### BV and Complications

BV is associated with the replacement of healthy vaginal lactobacilli by polymicrobial biofilms of select pathogens, including dominance by *Gardnerella vaginalis* [14] and cohabitation by other bacteria like *Atopobium vaginae*, *Mobiluncus* spp., *Bacteroides* spp., and *Prevotella* spp. [15]. A healthy woman’s vaginal microbiome (VMB) is dominated at ~95% by acid-producing bacteria that maintain a lower pH of <4.5 and help maintain vaginal epithelial barrier integrity [53], keeping other pathogens at bay. BV is marked by a shift from a *Lactobacillus*-dominant healthy vaginal microbiome comprising *L. crispatus*, *L. jensenni* and *L. gasseri*, which ensures this low pH and homeostasis, to a dysbiotic condition with an overgrowth of facultative and strict anaerobes and a higher pH [12], resulting in the remodeling of the vaginal environment and predisposition to secondary infections [13]. BV affects over 21 million women in the US alone, from the ages of 15 to 49 years [16]. Despite its association with important public health issues, including preterm labor, acquisition and transmission of sexually transmitted infections (STIs) and HIV [5,6], and associations with UTIs [7,8] and pelvic inflammatory disease [9], the pathogenesis of BV remains unclear [10,11]. One major outcome of recurrent BV is relevant to a generational impact, in that women with BV can have several reproductive complications, including but not limited to miscarriages (including 2nd trimester miscarriages) [54] and preterm births, often resulting in very low-birth-weight infants with increased susceptibility to neonatal sepsis [55]. While BV is much more commonly known to impact women, a study led by Damke and colleagues from Brazil [56] has indicated that seminal parameters could be affected by key markers of BV, including the identification of *G. vaginalis* during semen analysis, and a finding that 13 key markers of BV are commonly present (63.8%) in the semen of males seeking fertility evaluation, mainly *G. vaginalis,* at 50.7%. Another independent study showed that *G. vaginalis* colonizes semen in men attending infertility clinics in proportions of ~44% [57], and lastly, that a predominance of *G. vaginalis* in the female partner is related to significant inflammation in the male genital tract [58]. BV can be triggered by sex, multiple male or female partners [59], and practices like douching [60], and its prevalence can increase among women with copper-containing Intrauterine Device (IUD) [61]. Until recently, BV itself was not classified as an STI. A recent study pointed out that when male partners were included in antimicrobial therapy using combined oral and topical treatments, BV treatments resulted in a lower rate of recurrence within 12 weeks than standard care [62]. This crucial outcome may therefore shift treatment regimens in the foreseeable future. Current SOC antibiotics include Metronidazole and Clindamycin, administered via oral and topical vaginal routes, but these are unable to completely resolve the infection in ~70% of patients, resulting in high rates of recurrence. In fact, in many parts of the world, Metronidazole is the only SOC available to patients in need.

AMPs are proving to be an exciting area of research as alternatives to traditional antibiotics; however, there have been very few clinically successful AMP-based drugs, primarily due to issues of half-life, specificity, and toxicity [63]. Using a biological strategy, we have focused on human-milk-inspired AMPs, and have identified at least two AMPs, UCD-MAT-002 and MAT-006, as select therapeutic candidate(s). These AMPs are attractive therapeutic candidates, in part, because of their unique properties that circumvent issues critical to BV and are well-suited to the requirements of BV as an indication. (1) These AMPs are being developed for topical application for BV, and as such, extended systemic exposure is not a requirement for impact on pathogens. (2) The selected AMPs are potent against BV pathogens and inert against healthy vaginal lactobacilli, while still being efficacious for the transitional species, *L. iners*. (3) These AMPs are stable, not toxic to eukaryotic host cells, and their presence has the potential to reduce inflammation in the vaginal epithelium, which is associated with dysbiosis. These diverse, beneficial properties position these milk-inspired peptides as ideal candidates for the treatment of BV that may overcome the shortcomings of current therapeutic options. Importantly, unlike antibiotics that must enter the bacterial cell to have an impact at the DNA or protein synthesis level, and which can be slow-acting, AMPs rapidly eliminate pathogens via surface electrostatic interactions, making the development of resistance mechanistically and statistically less likely. Lastly, it is well known that BV is marked by a deficiency in AMPs, amongst other innate factors [64], resulting in further predisposition to other secondary infections [65], and hence, the approach of providing direct-to-site AMPs appears deliverable. One limitation of this study is the lack of AMP efficacy *in vivo*. Animal models for bacterial vaginosis do not fully recapitulate the complexity of humans [66] and limit therapeutic development. However, given the caveats of clinical development, where *in vivo* efficacy may not always translate to success in human trials, combinatorial approaches that combine *ex vivo* efficacy in the presence of diverse microbiomes and mucin composition and *in vivo* safety can foster preclinical de-risking of novel therapeutics.

Thus, in work to be described elsewhere, we show that these AMPs are efficacious ex vivo, as demonstrated by a significant reduction in the CFU load in vaginal samples obtained from patients symptomatic for BV through an IRB-approved study, and that both lead candidate human-milk-inspired AMPs, UCD-MAT-002 and MAT-006, can be formulated into vaginal inserts used in rabbit vaginal irritation studies. These inserts exhibit in vivo safety, even at AMP concentrations as high as 20%. Thus, the outcomes described herein can guide the development of a first-in-class topical vaginal insert as a novel therapeutic strategy to deliver human-milk-inspired AMPs into the vaginal milieu as a long-term solution for BV.

## Figures and Tables

**Table 1:** Bactericidal activity of human-milk-inspired AMPs on pathogens associated with BV. Four peptides, namely UCD-MAT-001, UCD-MAT-002, MAT-006 and MAT-014, were selected, with MBC values of 32 μg/mL or less, for the pathogens, and were tested using standard CLSI methods, as shown in the table. Healthy vaginal lactobacilli were included in the screen, and unlike the other three AMPs, MAT-014 was bactericidal to *L. crispatus*, *L. jensenni* and *L. gasseri*. Metronidazole (MET) served as a control in the assays.

***G. vaginalis* strains**	**MBC (μg/m** **L** **)** **—** **CLSI Method**				
	**MET**	**UCD-MAT-001**	**UCD-MAT-002**	**MAT-006**	**MAT-014**
14018 (ATCC)	4	32	16	16	8
HM-1105	64	16	8	8	4
HM-1106	32	8	8	8	4
HM-1107	32	32	16	8	8
HM-1110	64	32	16	8	8
HM-1112	32	16	8	8	8
**Other BV Associated Bacteria**	**MBC (μg/m** **L** **)** **—** **Time Kill Assays**				
	**MET**	**UCD-MAT-001**	**UCD-MAT-002**	**MAT-006**	**MAT-014**
*L. iners*	>500	16	8	8	8
*A. vaginae*	256	4	4	4	4
*M. indolicus*	4	8	8	8	4
*M. curtusii*	128	16	8	8	4
*L. crispatus*	>500	>500	>500	>500	32
*L. jensenii*	>500	>500	>500	>500	32
*L. gasserii*	>500	>500	>500	>500	32

**Table 2:** Selective activity of AMPs towards pathogens in a co-culture with beneficial *lactobacilli*. Equivalent CFUs of pathogenic and commensal bacteria were mixed and treated with AMPs in 0.8% NaCl or simulated vaginal fluid, and all AMPs but MAT-014 showed selective activity, indicated by “+” for activity (≥2 log reduction in CFU), and “−” for no activity (≤0.3 log reduction in CFU).

AMPs	BV Pathogens Blood Agar, HBT Bilayer, Tryptic Soy Agar, Brucella Agar	*Lactobacilli* (Beneficial Bacteria) MRS Agar
UCD-MAT-001	+(≥2.67 log reduction)	−
UCD-MAT-002	+(≥2.84 log reduction)	−
MAT-006	+(≥3.02 log reduction)	−
MAT-014	+(≥2.94 log reduction)	+(≥2.42 log reduction)

**Table 3:** The rate of the emergence of resistance to AMPs was measured using standard broth dilution methods starting at 1/20th the MBC for MAT-006 (shown here) and appears to be very unlikely and low.

Pathogen	CFU in the Absence of AMP	CFU in the Presence of AMP (MAT-006)	Rate of Resistance Emergence
*G. vaginalis*	2.67 × 10^9^	0	>1/2.67 × 10^9^
*A. vaginae*	2.9 × 10^9^	0	>1/2.9 × 10^9^
*M. curtusii*	2.45 × 10^9^	0	>1/2.45 × 10^9^

**Table 4:** All four AMPs were incubated at RT for the indicated times and evaluated for biological function via MBC assays. The AMPs were also exposed to higher temperatures of 42 and 70 °C prior to MBC assays. A “+” indicates that the AMP retained efficacy similar to that of the AMP stock solutions stored at −20 °C.

AMP	RT-3 Months	42 °C (1 h)	70 °C (1 h)
UCD-MAT-001	+	+	+
UCD-MAT-002	+	+	+
MAT-006	+	+	+
MAT-014	+	+	+

## Data Availability

The original contributions presented in this study are included in the article/Supplementary Material. Further inquiries can be directed to the corresponding author.

## Supplementary Materials

The following supporting information can be downloaded at: https://www.mdpi.com/article/10.3390/pharmaceutics18030371/s1, Figure S1: UCD-MAT-001 MS file; Figure S2: UCD-MAT-001 HPLC file; Figure S3: UCD-MAT-001 CoA; Figure S4: UCD-MAT-002 MS file; Figure S5: UCD-MAT-002 HPLC file; Figure S6: UCD-MAT-002 CoA; Figure S7: MAT-006 MS file; Figure S8: MAT-006 HPLC file; Figure S9: MAT-006 CoA; Figure S10: MAT-014 MS file; Figure S11: MAT-014 HPLC file; Figure S12: MAT-014 CoA; Figure S13: IND MS file; Figure S14: IND HPLC file; Figure S15: IND CoA file.
