# Supplementary material for: Selective Human-Milk-Inspired Antimicrobial Peptides for the Treatment of Bacterial Vaginosis
Source: Pharmaceutics. 2026 Mar 17;18(3):371. doi: 10.3390/pharmaceutics18030371 (PMC13029068; doi:10.3390/pharmaceutics18030371)
Supplement: Supplementary file 1 [file pharmaceutics-18-00371-s001.zip › pharmaceutics-4163224-supplementary.pdf]

Figure S1. UCD-MAT-001 MS File

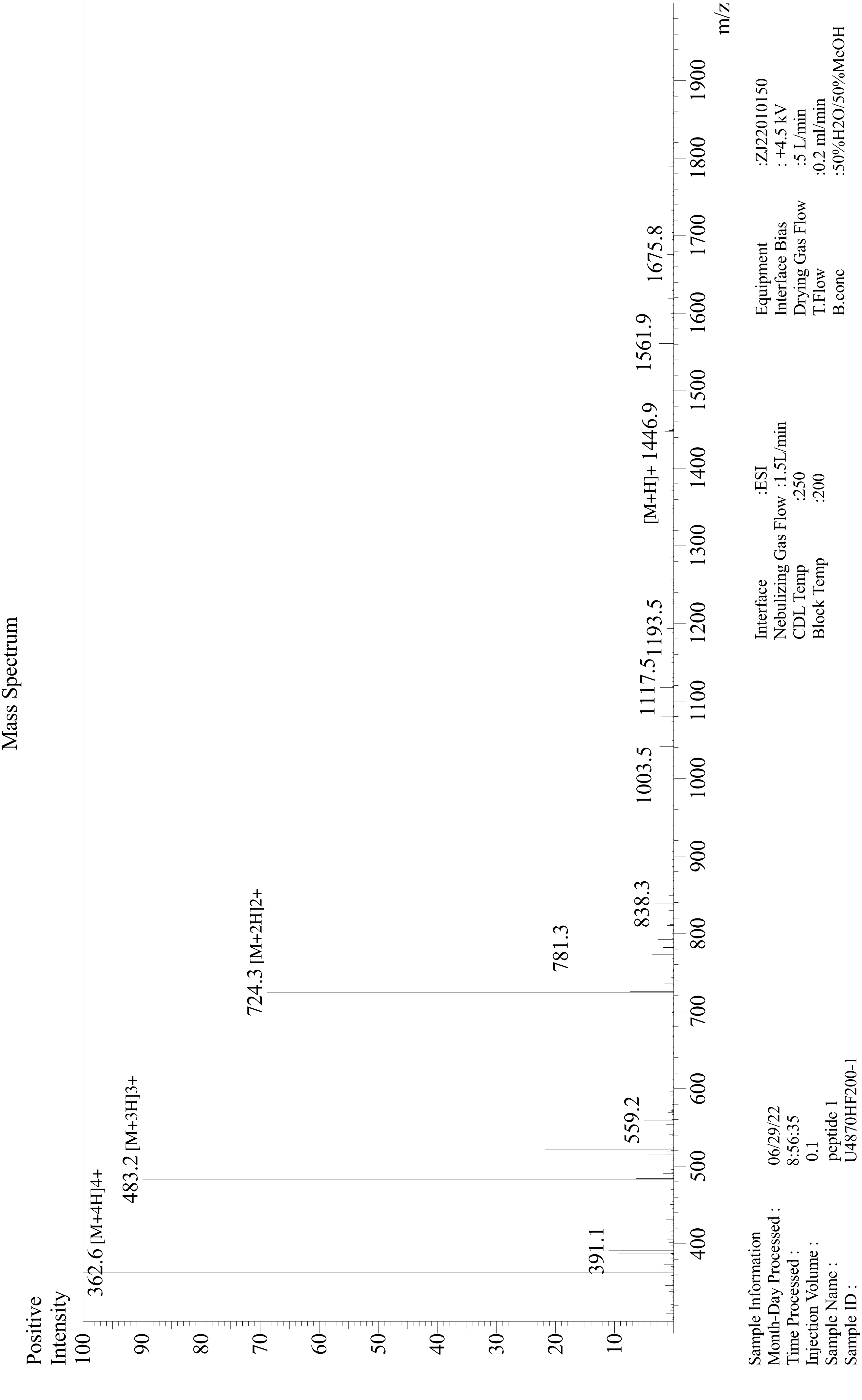

Figure S2. UCD-MAT-001 HPLC File

Sample Name :peptide 1
Sample ID :U4870HF200-1
Time Processed :12:51:16
Month-Day-Year Processed :07/05/2022

Pump A : 0.065% trifluoroacetic in 100% water (v/v)
Pump B : 0.05% trifluoroacetic in 100% acetonitrile (v/v)
Total Flow:1 ml/min
Wavelength:220 nm
<<LC Time Program>>

Time	Module	Command	Value
0.01	Pumps	B.Conc	5
25.00	Pumps	B.Conc	65
25.01	Pumps	B.Conc	95
37.00	Pumps	B.Conc	95
37.01	Pumps	B.Conc	95
45.00	Pumps	B.Conc	95
45.00	Controller	Stop	

<<Column Performance>>
<Detector A>
Column :Inertsil ODS-3 4.6 x 250 mm
Equipment: ZJ19010324

<Chromatogram>

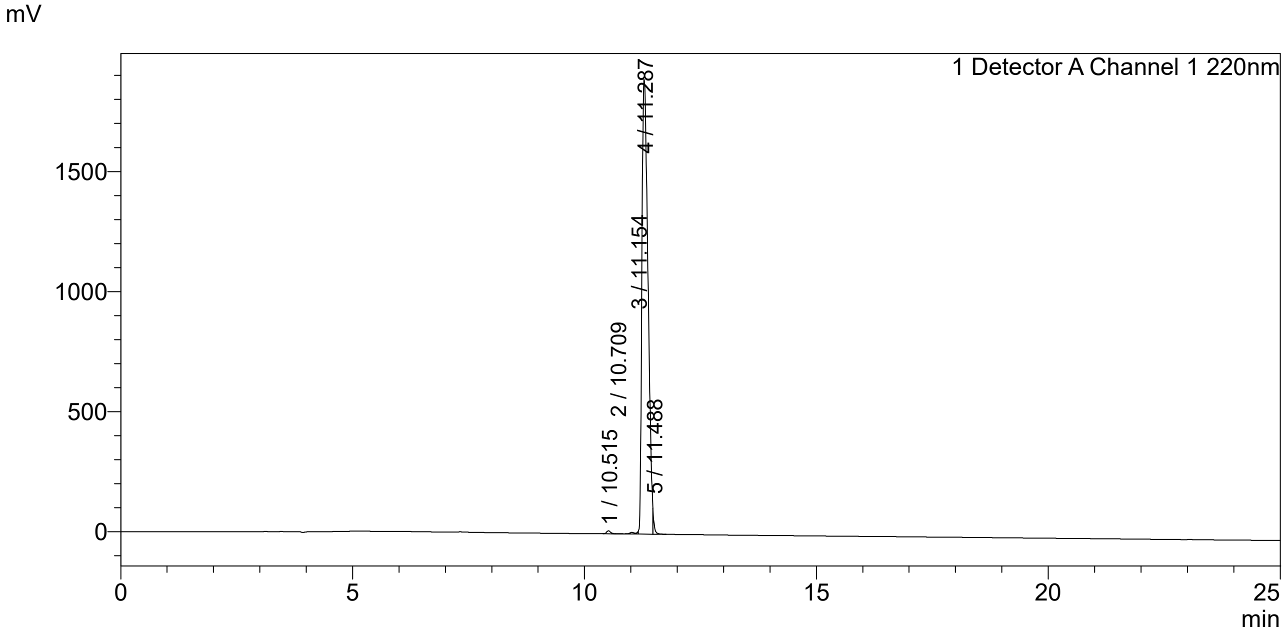

<Peak Table>

Detector A Channel 1 220nm

Peak#	Ret. Time	Area	Height	Area%
1	10.515	78775	12465	0.449
2	10.709	13426	1533	0.076
3	11.154	69551	8946	0.396
4	11.287	17236669	1892964	98.159
5	11.488	161611	82022	0.920
Total		17560032	1997930	100.000

Figure S3. UCD-MAT-001 COA

GenScript   Make Research Easy

CERTIFICATE OF ANALYSIS

**Product Name**	peptide 1
**Order ID**	U4870HF200_1
**Lot No.**	U4870HF200-1/PE4258
[redacted]	
**Modification**	N/A
**Length**	12AA
**Storage**	-20°C
**Recommended Solvent***	Ultrapure water
**Comments**	acetate salt

**Test Items**	**Specifications**	**Results**
**Molecular Weight**	Theoretical MW: 1446.79	Consistent
**HPLC purity**	≥95.0%	98.2%
**Appearance**	White crystal	Conforms
**Gross Weight**	150mg	150.9mg

*Note: Above recommended solvents for reference only. If there is any request for detailed dissolution conditions,we suggest you choose our 'Peptide Solubility Test Service'.

Caution:
For laboratory or further manufacturing use only. Not intended for household use. If you have any questions about the Certificate of Analysis, please contact our customer service representative at 1-877-436-7274 (Toll-Free), or 1-732-885-9188.

Certified by: Ni hui Wei   Date:07/13/2022

Thank you for your patronage to our Peptide services! To maintain this working relationship, we shall be grateful if you can add our webpage URL into your lab website. As a token of appreciation, you will be rewarded by 1,000 EZcoupon™ points. For more information, please contact us by e-mail at web@genscript.com

860 Centennial Ave., Piscataway, NJ 08854, USA

Toll-Free: 1-877-436-7274   Tel: 1-732-885-9188   Fax: 1-732-210-0262   Email: order@genscript.com   Web: www.genscript.com

Figure S4. UCD-MAT-002 MS File

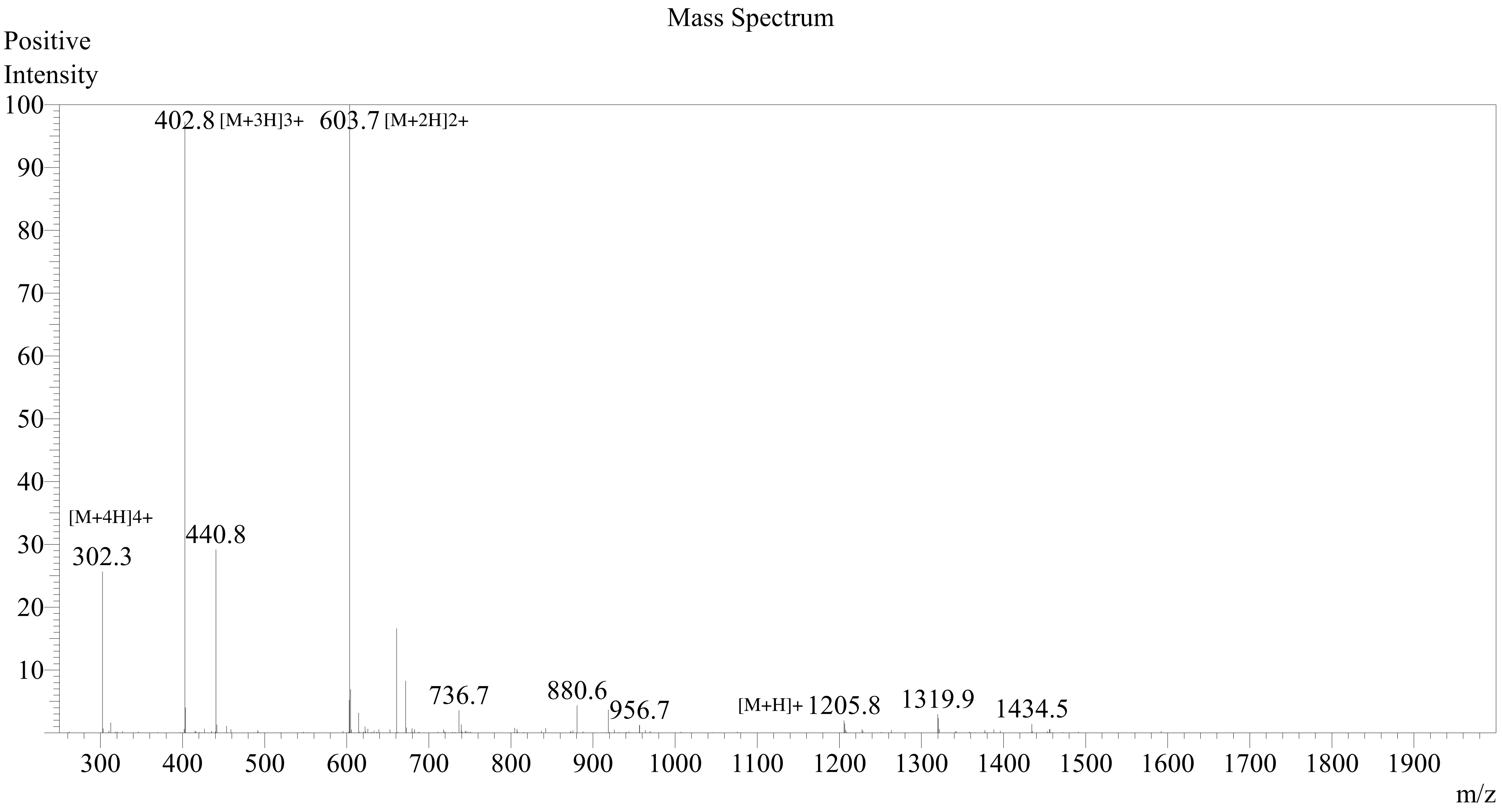

Sample Information	
Month-Day Processed :	06/29/22
Time Processed :	7:25:33 PM
Injection Volume :	0.1
Sample Name :	peptide 2
Sample ID :	U4870HF200-4
Theoretical MW :	1205.51
Observed MW :	1205.4

Interface	:ESI
Nebulizing Gas Flow	:1.5L/min
CDL Temp	:250
Block Temp	:200

Equipment	: ZJ21010035
Interface Bias	: +4.5 kV
Drying Gas Flow	:5 L/min
T.Flow	:0.2 ml/min
B.conc	:50%H2O/50%M

Figure S5. UCD-MAT-002 HPLC File

Sample Name :peptide 2
Sample ID :U4870HF200-4
Time Processed :11:59:47
Month-Day-Year Processed :06/29/2022

Pump A : 0.065% trifluoroacetic in 100% water (v/v)
Pump B : 0.05% trifluoroacetic in 100% acetonitrile (v/v)
Total Flow:1 ml/min
Wavelength:220 nm
<<LC Time Program>>

Time	Module	Command	Value
0.01	Pumps	B.Conc	5
25.00	Pumps	B.Conc	65
25.01	Pumps	B.Conc	95
27.00	Pumps	B.Conc	95
27.01	Pumps	B.Conc	5
35.00	Pumps	B.Conc	5
35.01	Controller	Stop	

<<Column Performance>>
<Detector A>
Column :Inertsil ODS-SP 4.6 x 250 mm
Equipment: ZJ21010026

<Chromatogram>

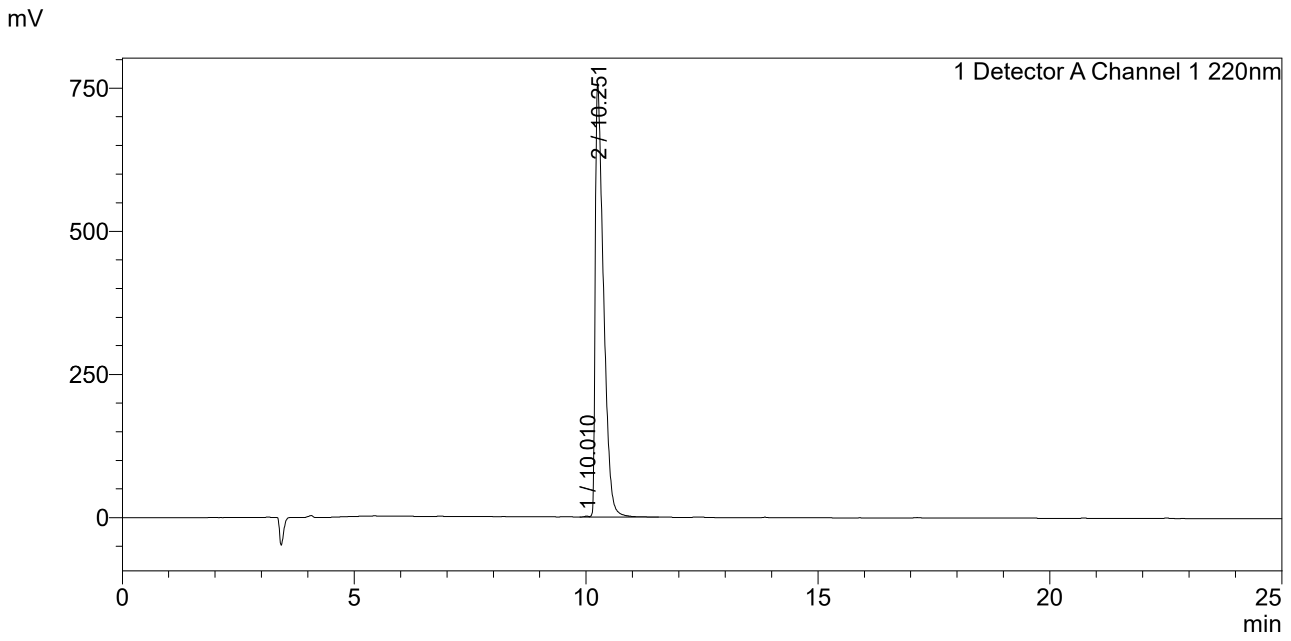

<Peak Table>

Detector A Channel 1 220nm

Peak#	Ret. Time	Area	Height	Area%
1	10.010	10531	1730	0.112
2	10.251	9355703	753613	99.888
Total		9366234	755342	100.000

Figure S6. UCD-MAT-002 COA

CERTIFICATE OF ANALYSIS

**Product Name**	peptide 2
**Order ID**	U4870HF200_4
**Lot No.**	U4870HF200-4/PE4261
[redacted]	
**Modification**	N/A
**Length**	10AA
**Storage**	-20°C
**Recommended Solvent***	Ultrapure water
**Comments**	acetate salt

**Test Items**	**Specifications**	**Results**
**Molecular Weight**	Theoretical MW: 1205.51	Consistent
**HPLC purity**	≥95.0%	99.9%
**Appearance**	White lyophilized powder	Conforms
**Gross Weight**	150mg	150.9mg

*Note: Above recommended solvents for reference only. If there is any request for detailed dissolution conditions,we suggest you choose our 'Peptide Solubility Test Service'.

Caution:
For laboratory or further manufacturing use only. Not intended for household use. If you have any questions about the Certificate of Analysis, please contact our customer service representative at 1-877-436-7274 (Toll-Free), or 1-732-885-9188.

Certified by: Xiaoqin Gao Date:07/02/2022

Thank you for your patronage to our Peptide services! To maintain this working relationship, we shall be grateful if you can add our webpage URL into your lab website. As a token of appreciation, you will be rewarded by 1,000 EZcoupon™ points. For more information, please contact us by e-mail at web@genscript.com

Figure S7. MAT-006 MS File

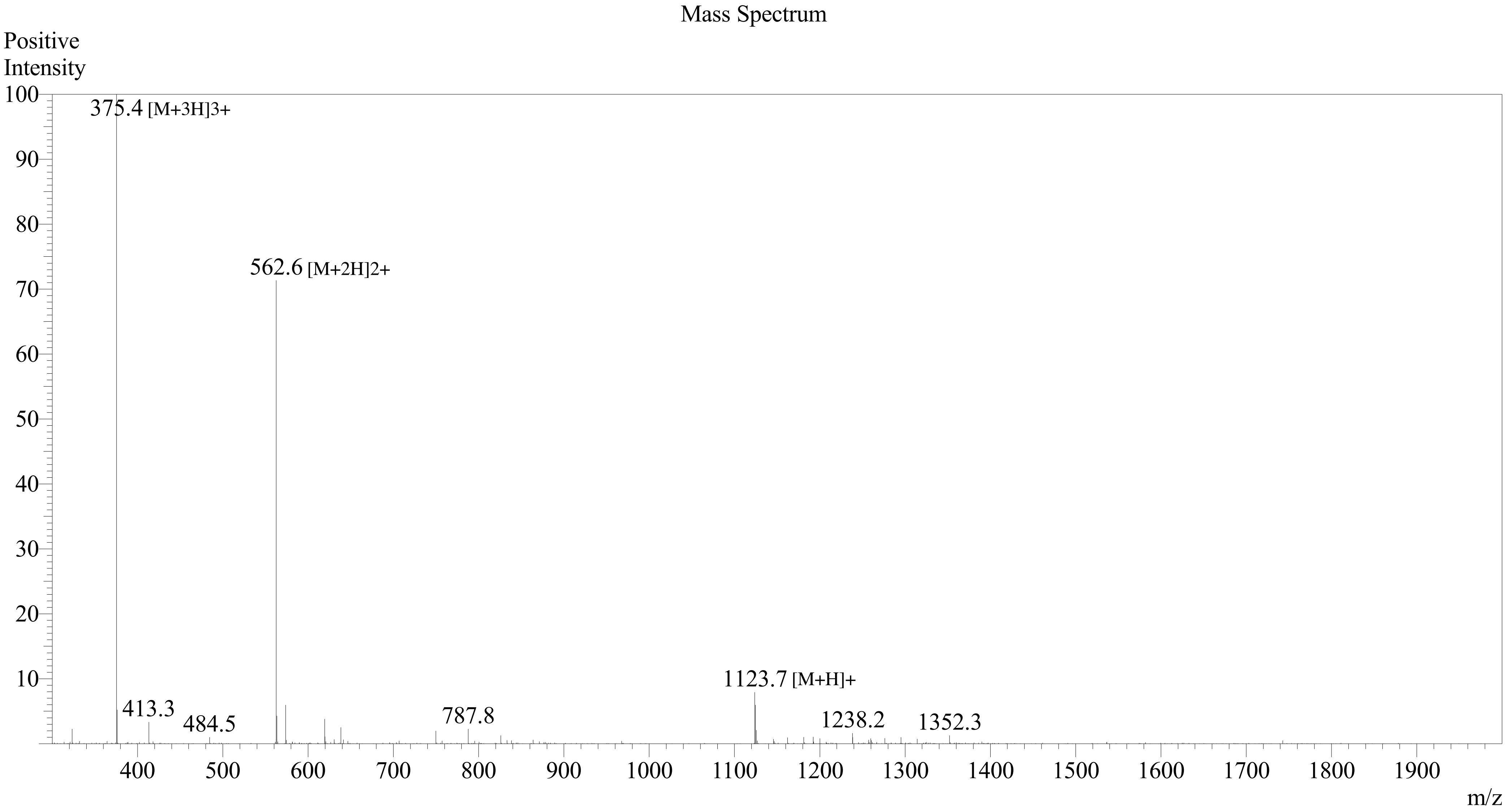

Sample Information	
Month-Day Processed :	06/29/22
Time Processed :	9:03:10
Injection Volume :	0.1
Sample Name :	peptide 3
Sample ID :	U4870HF200-7
Theoretical MW :	1123.36
Observed MW :	1123.2

Interface	:ESI
Nebulizing Gas Flow	:1.5L/min
CDL Temp	:250
Block Temp	:200

Equipment	:ZJ22010150
Interface Bias	: +4.5 kV
Drying Gas Flow	:5 L/min
T.Flow	:0.2 ml/min
B.conc	:50%H2O/50%MeOH

Figure S8. MAT-006 HPLC File

Sample Name :peptide 3
Sample ID :U4870HF200-7
Time Processed :2:08:23 AM
Month-Day-Year Processed :06/29/2022

Pump A : 0.065% trifluoroacetic in 100% water (v/v)
Pump B : 0.05% trifluoroacetic in 100% acetonitrile (v/v)
Total Flow:1 ml/min
Wavelength:220 nm
<<LC Time Program>>

Time	Module	Command	Value
0.01	Pumps	B.Conc	5
25.00	Pumps	B.Conc	65
25.01	Pumps	B.Conc	95
27.00	Pumps	B.Conc	95
27.01	Pumps	B.Conc	5
35.00	Pumps	B.Conc	5
35.01	Controller	Stop	

<<Column Performance>>
<Detector A>
Column :Inertsil ODS-SP 4.6 x 250 mm
Equipment: ZJ21010376

<Chromatogram>

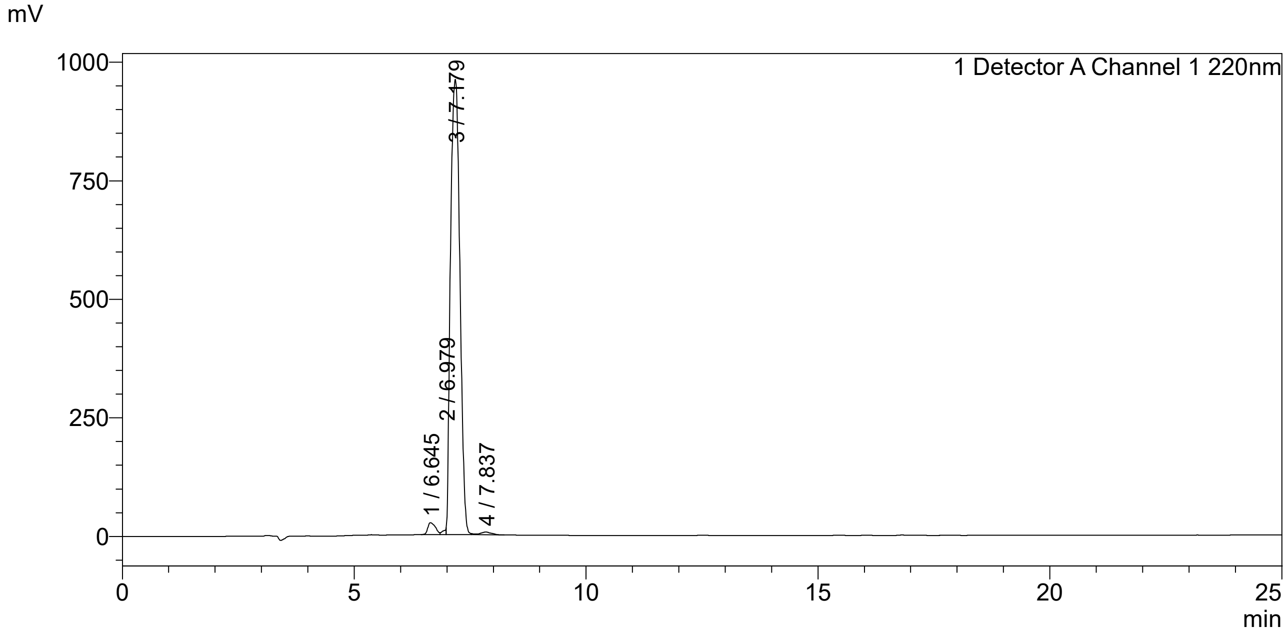

<Peak Table>

Detector A Channel 1 220nm

Peak#	Ret. Time	Area	Height	Area%
1	6.645	288588	25217	2.085
2	6.979	61702	10655	0.446
3	7.179	13392637	960250	96.756
4	7.837	98793	5707	0.714
Total		13841721	1001829	100.000

Figure S9. MAT-006 COA

CERTIFICATE OF ANALYSIS

**Product Name**	peptide 3
**Order ID**	U4870HF200_7
**Lot No.**	U4870HF200-7/PE4264
[redacted]	
**Modification**	N/A
**Length**	10AA
**Storage**	-20°C
**Recommended Solvent***	Ultrapure water
**Comments**	acetate salt

**Test Items**	**Specifications**	**Results**
**Molecular Weight**	Theoretical MW: 1123.36	Consistent
**HPLC purity**	≥95.0%	96.8%
**Appearance**	White crystal	Conforms
**Gross Weight**	150mg	150.0mg

*Note: Above recommended solvents for reference only. If there is any request for detailed dissolution conditions,we suggest you choose our 'Peptide Solubility Test Service'.

Caution:
For laboratory or further manufacturing use only. Not intended for household use. If you have any questions about the Certificate of Analysis, please contact our customer service representative at 1-877-436-7274 (Toll-Free), or 1-732-885-9188.

Certified by: Yi Gao Date:07/01/2022

Thank you for your patronage to our Peptide services! To maintain this working relationship, we shall be grateful if you can add our webpage URL into your lab website. As a token of appreciation, you will be rewarded by 1,000 EZcoupon™ points. For more information, please contact us by e-mail at web@genscript.com

Figure S10. MAT-014 MS File

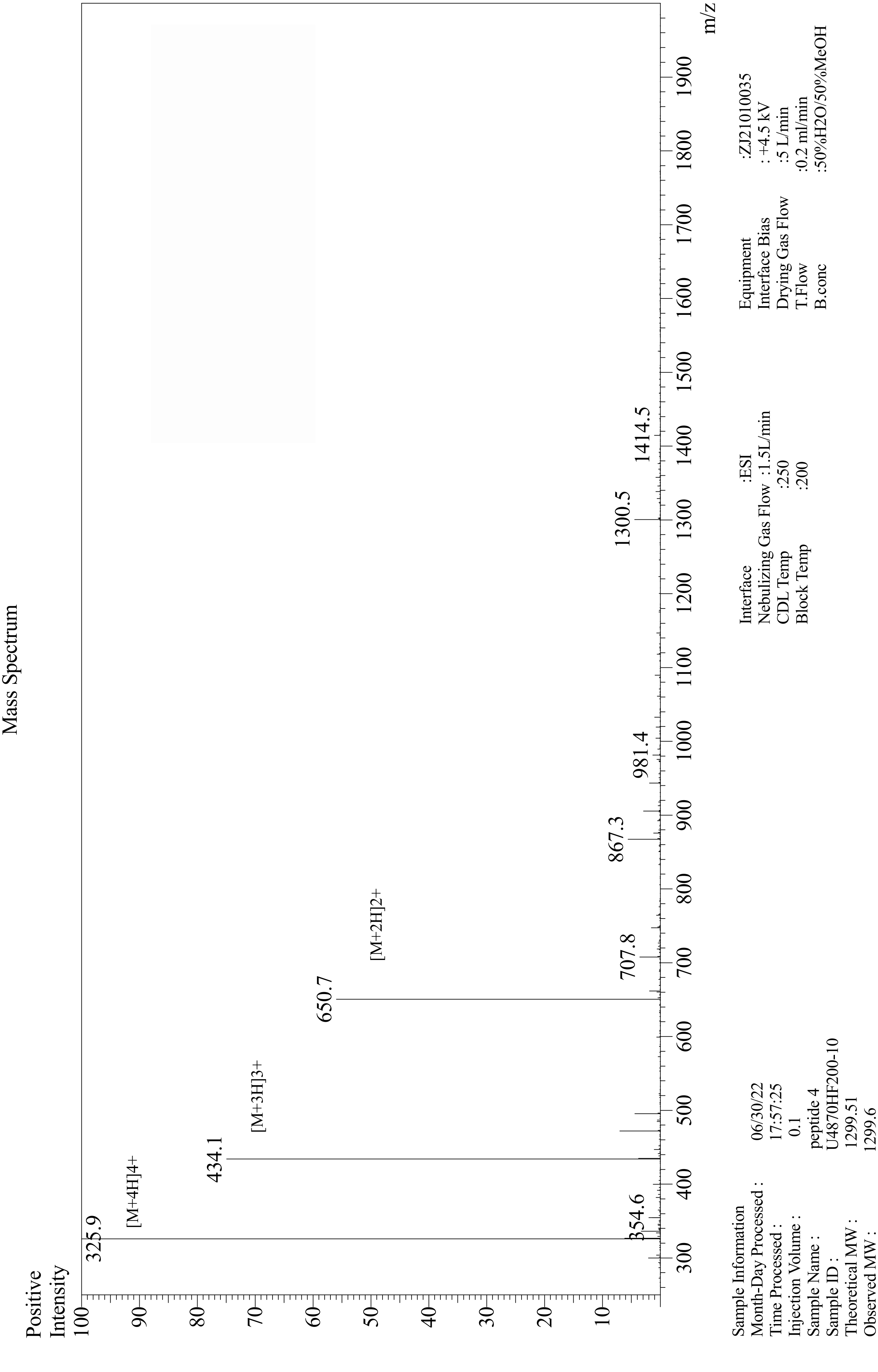

Figure S11. MAT-014 HPLC File

Sample Name :peptide 4
Sample ID :U4870HF200-10
Time Processed : 8:17:11
Month-Day-Year Processed :07/01/2022

Pump A : 0.065% trifluoroacetic in 100% water (v/v)
Pump B : 0.05% trifluoroacetic in 100% acetonitrile (v/v)
Total Flow:1 ml/min
Wavelength:220 nm
<<LC Time Program>>

Time	Module	Command	Value
0.01	Pumps	Solvent B Conc.	5
25.00	Pumps	Solvent B Conc.	65
25.01	Pumps	Solvent B Conc.	95
27.00	Pumps	Solvent B Conc.	95
27.01	Pumps	Solvent B Conc.	5
33.00	Pumps	Solvent B Conc.	5
33.01	Controller	Stop	

<<Column Performance>>
<Detector A>
Column : Inertsil ODS-SP 4.6 x 250 mm
Equipment:ZJ17010507

<Chromatogram>

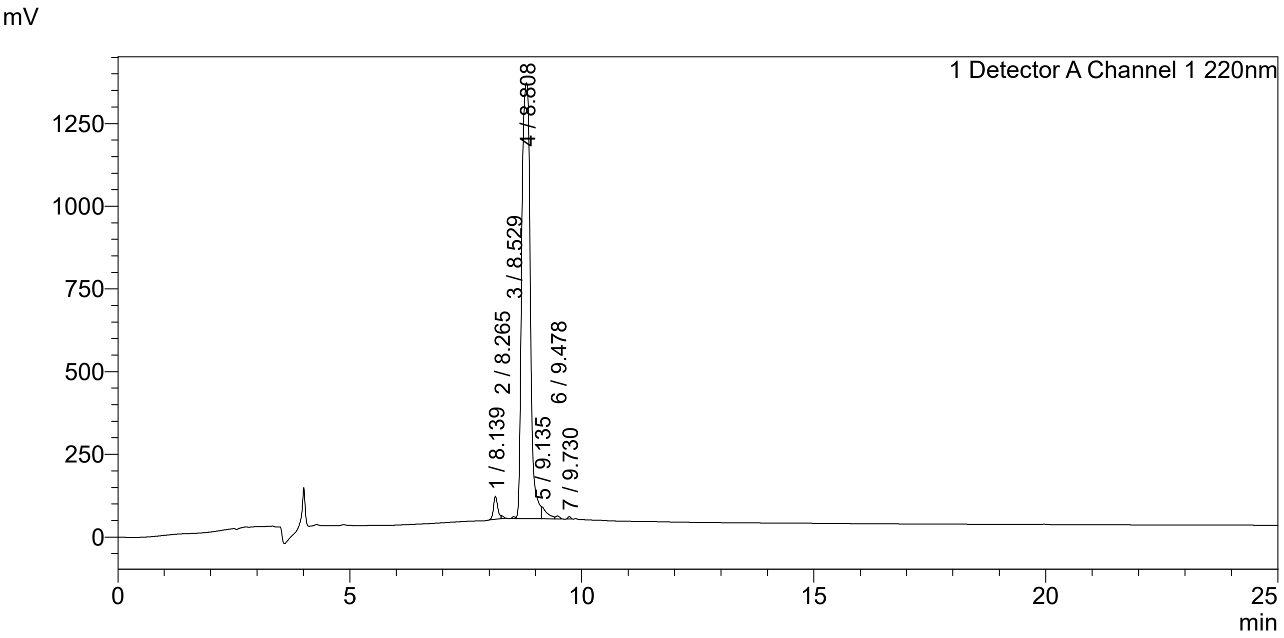

<Peak Table>

Detector A Channel 1 220nm

Peak#	Ret. Time	Area	Height	Area%
1	8.139	441925	69451	2.503
2	8.265	31055	10015	0.176
3	8.529	26011	5427	0.147
4	8.808	16778389	1318100	95.048
5	9.135	295055	37438	1.671
6	9.478	54246	9210	0.307
7	9.730	25857	6590	0.146
Total		17652538	1456231	100.000

Figure S12. MAT-014 COA

CERTIFICATE OF ANALYSIS

**Product Name**	peptide 4
**Order ID**	U4870HF200_10
**Lot No.**	U4870HF200-10/PE4267
[redacted]	
**Modification**	N/A
**Length**	11AA
**Storage**	-20°C
**Recommended Solvent***	Ultrapure water
**Comments**	acetate salt

**Test Items**	**Specifications**	**Results**
**Molecular Weight**	Theoretical MW: 1299.51	Consistent
**HPLC purity**	≥95.0%	95.0%
**Appearance**	White lyophilized powder	Conforms
**Gross Weight**	150mg	150.0mg

*Note: Above recommended solvents for reference only. If there is any request for detailed dissolution conditions,we suggest you choose our 'Peptide Solubility Test Service'.

Caution:
For laboratory or further manufacturing use only. Not intended for household use. If you have any questions about the Certificate of Analysis, please contact our customer service representative at 1-877-436-7274 (Toll-Free), or 1-732-885-9188.

Certified by: Sun ting wang Date:07/03/2022

Thank you for your patronage to our Peptide services! To maintain this working relationship, we shall be grateful if you can add our webpage URL into your lab website. As a token of appreciation, you will be rewarded by 1,000 EZcoupon™ points. For more information, please contact us by e-mail at web@genscript.com

860 Centennial Ave., Piscataway, NJ 08854, USA
Toll-Free: 1-877-436-7274 Tel: 1-732-885-9188 Fax: 1-732-210-0262 Email: order@genscript.com Web: www.genscript.com

Figure S13. IND MS File

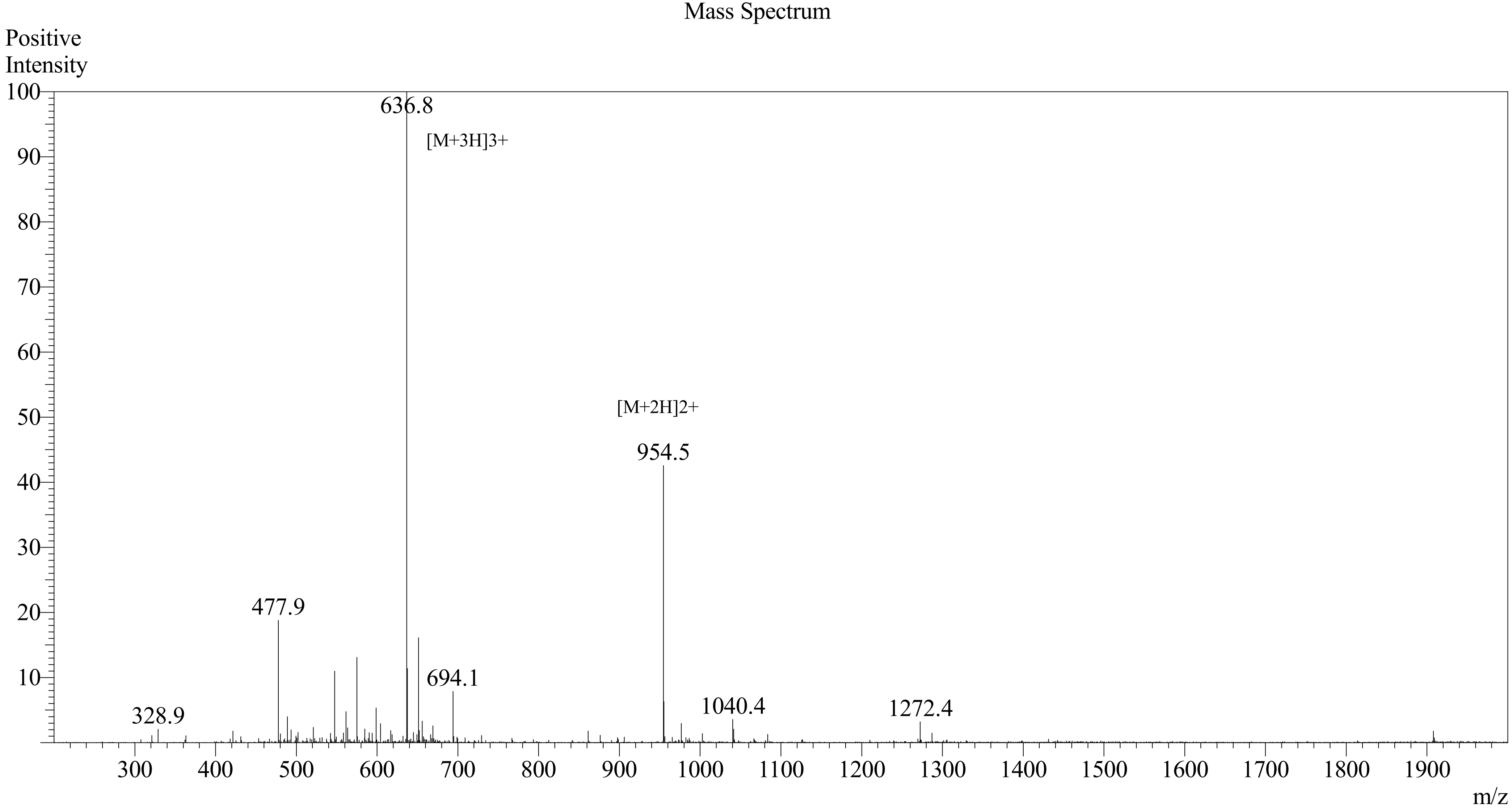

Sample Information	
Acquired by :	Gary
Month-Day Processed :	04/29/21
Time Processed :	9:57:25 PM
Injection Volume :	0.4
Sample Name :	11
Sample ID :	U339MGD230-11
Theoretical MW :	1907.28
Observed MW :	1907.4

Interface	:ESI
Nebulizing Gas Flow	:1.5L/min
CDL Temp	:250¡æ
Block Temp	:200¡æ

Equipment:	SS-CM-0273
Interface Bias:	+4.5 kV
Drying Gas Flow:	5 L/min
T.Flow:	0.2 ml/min
B.conc:	50%H2O/50%MeOH

Figure S14. IND HPLC File

Sample Name :11
Sample ID :U339MGD230-11
Time Processed :8:37:49 PM
Month-Day-Year Processed :05/02/2021

Pump A : 0.065% trifluoroacetic in 100% water (v/v)
Pump B : 0.05% trifluoroacetic in 100% acetonitrile (v/v)
Total Flow:1 ml/min
Wavelength:220 nm
<<LC Time Program>>

Time	Module	Command	Value
0.01	Pumps	Pump A B.Conc	5
25.00	Pumps	Pump A B.Conc	65
25.01	Pumps	Pump A B.Conc	95
27.00	Pumps	Pump A B.Conc	95
27.01	Pumps	Pump A B.Conc	5
35.00	Pumps	Pump A B.Conc	5
35.01	Controller	Stop	

<<Column Performance>>
<Detector A>
Column :Inertsil ODS-3 4.6 x 250 mm
Equipment: GR11010440

<Chromatogram>

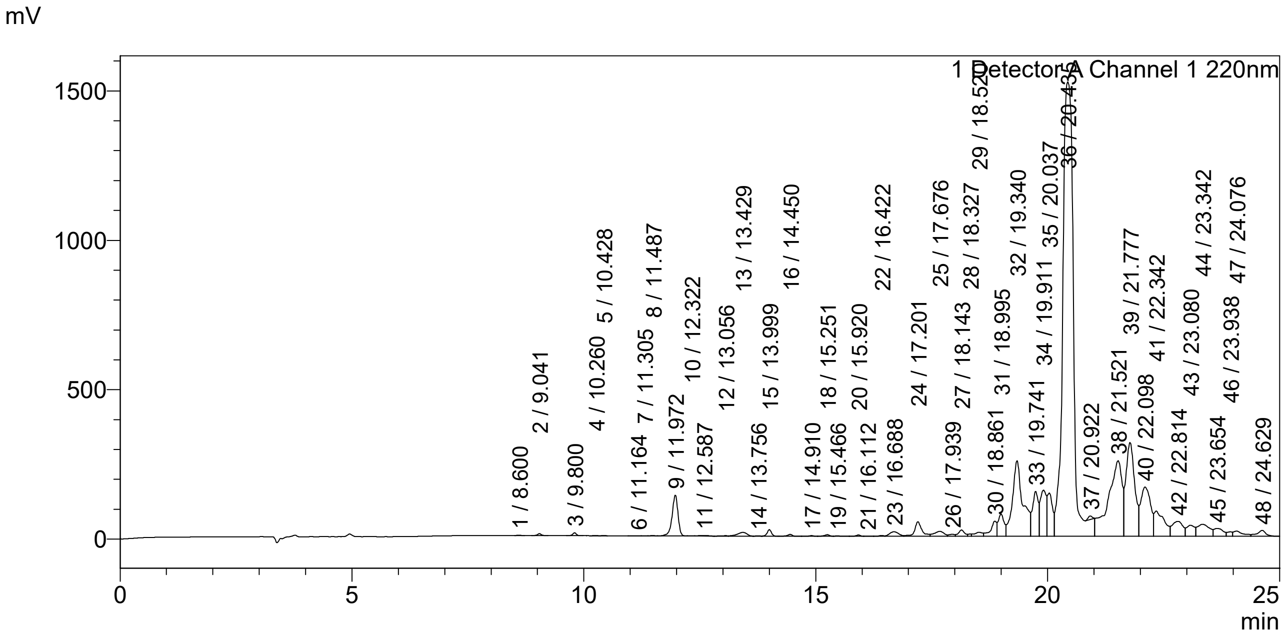

<Peak Table>

Detector A Channel 1 220nm

Peak#	Ret. Time	Area	Height	Area%
1	8.600	3917	705	0.007
2	9.041	36013	6199	0.069
3	9.800	54880	9523	0.104
4	10.260	3356	657	0.006
5	10.428	2700	500	0.005
6	11.164	2818	360	0.005
7	11.305	3154	575	0.006
8	11.487	7622	1201	0.015
9	11.972	1205371	136661	2.293
10	12.322	2253	421	0.004
11	12.587	26945	1997	0.051
12	13.056	10843	1494	0.021
13	13.429	177539	12486	0.338
14	13.756	3612	725	0.007
15	13.999	140852	22060	0.268
16	14.450	46741	6182	0.089

Peak#	Ret. Time	Area	Height	Area%
17	14.910	16083	2265	0.031
18	15.251	44285	5500	0.084
19	15.466	12042	1558	0.023
20	15.920	29977	4478	0.057
21	16.112	3063	316	0.006
22	16.422	21664	3051	0.041
23	16.688	210420	15429	0.400
24	17.201	531411	48905	1.011
25	17.676	247978	16575	0.472
26	17.939	58994	6596	0.112
27	18.143	197247	21241	0.375
28	18.327	36272	7375	0.069
29	18.520	164458	13427	0.313
30	18.861	460095	50539	0.875
31	18.995	659220	75241	1.254
32	19.340	3968322	253252	7.550
33	19.741	1312608	150417	2.497
34	19.911	1401457	154661	2.666
35	20.037	1136633	144727	2.162
36	20.435	24173653	1521890	45.990
37	20.922	72692	11510	0.138
38	21.521	5249561	252963	9.987
39	21.777	4142277	313990	7.881
40	22.098	2439832	164536	4.642
41	22.342	1290992	84528	2.456
42	22.814	798505	49836	1.519
43	23.080	443178	36839	0.843
44	23.342	734505	40014	1.397
45	23.654	361845	25837	0.688
46	23.938	118323	14697	0.225
47	24.076	245755	17280	0.468
48	24.629	250472	19669	0.477
Total		52562431	3730886	100.000

Figure S15. IND COA

CERTIFICATE OF ANALYSIS

**Product Name**	11
**Order ID**	U339MGD230_11
**Lot No.**	U339MGD230-11/PE3007
[redacted]	
**Modification**	N/A
**Length**	13AA
**Storage**	-20°C
**Recommended Solvent***	ultrapure water
**comments**	TFA salt

Test Items	Specifications	Results
**Molecular Weight**	Theoretical MW: 1907.28	Consistent
**HPLC purity**	Crude	Crude
**Appearance**	White lyophilized powder	Conforms
**Gross Weight**	9 mg	9.8mg

*Note: Above recommended solvents for reference only. If there is any request for detailed dissolution conditions,we suggest you choose our 'Peptide Solubility Test Service'.

Caution:
For laboratory or further manufacturing use only. Not intended for household use. If you have any questions about the Certificate of Analysis, please contact our customer service representative at 1-877-436-7274 (Toll-Free), or 1-732-885-9188.

Certified by: Gary Chen Date:05-07-2021
Peptide Production Director

Thank you for your patronage to our Peptide services! To maintain this working relationship, we shall be grateful if you can add our webpage URL into your lab website. As a token of appreciation, you will be rewarded by 1,000 EZcoupon™ points. For more information, please contact us by e-mail at web@genscript.com
